# Supplementary material for: Resolving the full spectrum of human genome variation using Linked-Reads
Source: Genome Res. 2019 Apr;29(4):635–45. doi: 10.1101/gr.234443.118 (PMC6442396; doi:10.1101/gr.234443.118)
Supplement: Supplemental Material [file supp_gr.234443.118_Supplemental_longranger-2.2.zip › longranger-2.2.tar.bz2!/lr2.2-public/lib/stan/cmdstan/stan/doc/api/html/agrad_2rev_2matrix_2divide_8hpp_source.html]

Stan: src/stan/agrad/rev/matrix/divide.hpp Source File


|  |  |
| --- | --- |
|  | Stan  2.5.0  probability, sampling & optimization |


- Main Page
- Related Pages
- Namespaces
- Classes
- Files

- File List
- File Members

All Classes Namespaces Files Functions Variables Typedefs Enumerations Enumerator Friends Macros Pages

- src
- stan
- agrad
- rev
- matrix

divide.hpp

Go to the documentation of this file.

1 #ifndef STAN\_\_AGRAD\_\_REV\_\_MATRIX\_\_DIVIDE\_HPP

2 #define STAN\_\_AGRAD\_\_REV\_\_MATRIX\_\_DIVIDE\_HPP

3

4 #include <vector>

5 #include <stan/math/matrix/Eigen.hpp>

6 #include <stan/math/matrix/typedefs.hpp>

7 #include <stan/agrad/rev/var.hpp>

8 #include <stan/agrad/rev/operators/operator\_division.hpp>

9 #include <stan/agrad/rev/matrix/to\_var.hpp>

10 #include <stan/agrad/rev/matrix/typedefs.hpp>

11

12 namespace stan {

13  namespace agrad {

14

22  inline double

23  divide(double x, double y) {

24  return x / y;

25  }

26  template <typename T1, typename T2>

27  inline var

28  divide(const T1& v, const T2& c) {

29  return to\_var(v) / to\_var(c);

30  }

38  template <typename T1, typename T2, int R, int C>

39  inline Eigen::Matrix<var,R,C>

40  divide(const Eigen::Matrix<T1, R,C>& v, const T2& c) {

41  return to\_var(v) / to\_var(c);

42  }

43

44  }

45 }

46 #endif

typedefs.hpp

stan::agrad::to\_var

var to\_var(const double &x)

Converts argument to an automatic differentiation variable.

**Definition:** to\_var.hpp:21

operator\_division.hpp

typedefs.hpp

to\_var.hpp

var.hpp

Eigen.hpp

stan::agrad::divide

stan::return\_type< T1, T2 >::type divide(const T1 &v, const T2 &c)

**Definition:** divide.hpp:17


---

[ Stan Home Page ]

*© 2011–2014,
Stan Development Team.*
